# Biomass Grinding Process Optimization Using Response Surface Methodology and a Hybrid Genetic Algorithm

**DOI:** 10.3390/bioengineering6010012

**Published:** 2019-01-25

**Authors:** Jaya Shankar Tumuluru, Dean J. Heikkila

**Affiliations:** 1 Idaho National Laboratory, 750 MK Simpson Blvd., Energy Systems Laboratory, P.O. Box: 1625, Idaho Falls, ID 83415-3570, USA; 2 Intern, University of Washington, 1410 NE Campus Parkway, Seattle, WA 98195, USA; deanjheikkila@gmail.com

**Keywords:** renewable energy, corn stover, grinding process, optimization, response surface methodology, hybrid genetic algorithm

## Abstract

Biomass could be a key source of renewable energy. Agricultural waste products, such as corn stover, provide a convenient means to replace fossil fuels, such as coal, and a large amount of feedstock is currently available for energy consumption in the U.S. This study has two main objectives: (1) to understand the impact of corn stover moisture content and grinder speed on grind physical properties; and (2) develop response surface models and optimize these models using a hybrid genetic algorithm. The response surface models developed were used to draw surface plots to understand the interaction effects of the corn stover grind moisture content and grinder speed on the grind physical properties and specific energy consumption. The surface plots indicated that a higher corn stover grind moisture content and grinder speed had a positive effect on the bulk and tapped density. The final grind moisture content was highly influenced by the initial moisture content of the corn stover grind. Optimization of the response surface models using the hybrid genetic algorithm indicated that moisture content in the range of 17 to 19% (w.b.) and a grinder speed of 47 to 49 Hz maximized the bulk and tapped density and minimized the geomantic mean particle length. The specific energy consumption was minimized when the grinder speed was about 20 Hz and the corn stover grind moisture content was about 10% (w.b.).

## 1. Introduction

The social and environmental benefits of using carbon-neutral biomasses are resulting in its increased use for renewable biomaterials. According to the United States (U.S.) Department of Energy’s (DOE) Bioenergy Technologies Office (BETO), there is more than a billion tons of biomass available in the U.S. for bioenergy and biomaterial production. Among these vast biomass sources, wood and agricultural wastes are fast becoming the first choice as a renewable non-food source of the lignocellulosic biomaterial. According to the Food and Agriculture Organization of the United Nations (FAO) [1], 31% of the total land area on planet Earth is covered by forests [2].

Biomass is widely used as fuel, construction material, and raw material for biofuels and biobased products. According to Tumuluru [3], biomass preprocessing and pretreatments using mechanical, chemical, and thermal methods play a major role in improving the biomass physical, chemical, and thermal properties, and make it more suitable for solid and liquid fuels production. The same author has indicated that mechanical preprocessing methods, such as size-reduction and densification, help to improve biomass physical properties, such as moisture content, bulk and tapped density, and particle size distribution. In fact, one of the major unit operations in using biomass for many applications is grinding. Size-reduction or grinding helps to convert biomass from a non-flowable, packaged state to a more flowable feedstock with particle sizes suitable for both biochemical and thermochemical conversion processes.

Grinders are the major machines used in biomass harvesting systems. They are capital-intensive and have high throughputs [4]. The energy consumption of the grinder varies based on the type of biomass—woody, herbaceous, and municipal solid waste—and other biomass feedstock properties, such as moisture content and biomass composition. Grinder machine parameters, such as the type of the mill (e.g., shear, impact, attrition) and the screen size of the grinder also result in different energy consumptions [5]. Typically, the grinding energy for a hammer mill varies between 5 and 60 kWh/ton [6,7]. The type of the grinder is important for reducing energy input when preparing biomass. If the grinding device is a knife, its geometry and the direction of the cut in relation to the workpiece affects the configuration of the resulting chips, thereby cutting power requirements and the quality of the surface of the chip [8].

In general, size-reduction of woody and herbaceous biomass involves a two-stage grinding process. In the first stage, the grinder breaks the woody chips or herbaceous biomass bales into a larger size material, thereby making it more flowable in the conveyors. In the second stage of the grinding process, the biomass is further ground to a smaller size to make the biomass suitable for biochemical and thermochemical conversion processes. Typically for biochemical conversion, the biomass is ground in a hammer mill fitted with a 25.4 mm screen, whereas for thermochemical conversions, such as pyrolysis and gasification, the biomass is ground to smaller particle sizes, typically around 2 mm (i.e., 0.08 in). According to Dibble et al. [9] and van Walsum et al. [10], the smaller particle sizes help control the reaction kinetics and achieve the desired conversion efficiency in the thermochemical conversion processes.

Mechanical grinding of lignocellulosic substances, such as wood and corn stover, typically leads to a fine particle size, various particle shapes, high specific surface area, and sometimes low cellulose crystallinity [2], which depends on the grinding mechanism, grinding process conditions, and the type of raw material. Grinding the raw biomass into smaller particles has a great impact on the storage and conveying properties and its suitability for a given application. There are two types of grinding: coarse grinding and fine grinding. The raw biomass that is harvested from forests or fields goes through a coarse grinding process. This coarsely ground biomass is further ground to a fine size for various applications. However, the definition of fine grinding varies with the industrial application [11,12,13,14,15]. In the case of lignocellulosic biomasses, such as wood, the term “fine grinding” is used for product sizes less than 100 μm, whereas for other studies, fine grinding is used for product sizes up to 1 mm [16,17,18,19]. In the case of agriculture straws, crop residues, and wood for biofuels production, the raw material is initially ground using a Stage-1 grinder, which is typically fitted with a screen in the range of 50.8 to 152.4 mm (i.e., 2 to 6 in) and is considered a coarse grind. The coarse grind is further size-reduced to ≤25.4 mm (i.e., ≤1 in) in a Stage-2 grinder. The screen size used in the Stage-2 grinder depends on the conversion pathway selected. In the case of pelleting, a 6.4 mm (i.e., ¼ in) screen is typically used, whereas for thermochemical conversion applications, an even smaller screen of 2 mm (i.e., 0.08 in) is used.

### 1.1. Grinding Process Variables

For his Ph.D. thesis, Newbolt [20] collected information on the type of mill, fuel type, and comminution variables. His literature review indicated that the hammer mill is the most preferred for biomass size-reduction, whereas the cutting mill is the next most preferred. The frequently tested process variables are screen size, angular velocity, time, feed rate, type, feed size, load, moisture content, and process. The commonly measured dependent variable for the grinding process is particle size distribution, specific grinding energy, geometric mean particle size, moisture content, bulk density, absolute density, Rosin_Rammler Char, comminution ratio, uniformity index, percentiles (i.e., 10, 50, and 90), mass relative span, and aspect ratio. The literature review also indicated that particle size distributions and specific grinding energy are the prime important dependent variables that are measured for size-reduced biomass. The study by Tumuluru and Yancey [7] indicated that both the Stage-1 and Stage-2 grinding processes are influenced by process variables, such as biomass moisture content, screen size used in the grinder, grinder type, and feedstock type. According to Lopo [21], energy consumption of grinding biomass depends on initial particle size, moisture content, material properties, the feed rate of the material, and machine variables. Studies conducted by Mani et al. [22], Tavakoli et al. [23], and Holtzapple et al. [24] reported that there is an inverse relationship between grinding energy and screen size selected for both woody and herbaceous biomass. Vigneault et al. [25] indicated that the total specific energy of hammer mill grinding has a direct correlation to an increase in hammer tip speed. O’Dogherty [26] indicated that the feeding rate also has a significant effect on specific energy consumption during hammer mill grinding and has a positive correlation. Studies by Bitra et al. [27], Arthur et al. [28], and Himmel et al. [29] indicated that the total specific energy for knife mill and tub grinder is negatively correlated with the screen size and mass feed rate.

### 1.2. Response Surface Methodology

Response surface methodology (RSM) is a technique that is used to improve or optimize process performance [30]. In general process, optimization is conducted using statistical methods, such as RSM, or using evolutionary algorithms (EAs), such as genetic algorithms (GAs). Khuri and Mukhopadhyay [31] state that RSM consists of a combination of mathematical and statistical techniques used to develop a functional relationship between a response variable (y) with respect to the process variables tested (x_1_, x_2_, …, x_k_). Bezerra et al. [32] reviewed the application of RSM as a tool for optimization in analytical chemistry. They indicated that RSM helps to develop the desirability function, which can be further used for optimization. According to Kleijen [33], RSM is a stepwise heuristic that uses first-order polynomials to approximate the response surface locally.

RSM combines the design of experiments, regression analysis, and optimization methods in a general purpose strategy to optimize the expected value of a stochastic response. In their paper on product quality improvement through RSM, Zhen et al. [34] indicated that this technique is an important tool for product and process improvement. They stated that RSM has extensive applications where several input variables have a significant impact on process performance and quality characteristics. They also stated that the initial step of RSM is the design of experiments that help to determine the lower and upper limits for conducting the experiments. Several researchers have used RSM for model development and optimization. Francis et al. [35] used RSM for process parameter optimization for the production of α-amylase by *Aspergillus oryzae*. In their study, a Box–Behnken experimental design was used to optimize three process parameters—incubation temperature, initial substrate moisture, and inoculum size—for the optimal production of α-amylase by *Aspergillus oryzae* NRRL 6270 in solid-state fermentation (SSF). Quanhong and Caili [36] used RSM for extraction optimization on the germinant pumpkin seeds protein. The study was focused on trying to understand how the liquid-solid ratio, NaCl concentration, and reaction time impacted the protein production from the germinant pumpkin seeds. In their studies on the optimization of cutting conditions for surface roughness using RSM, Öktem et al. [37] used a combination of RSM and a GA for parameters such as feed, cutting speed, axial depth of cut, radial depth of cut, and machining tolerance. Optimization of the process using the GA has helped to reduce surface roughness from 0.412 mm to 0.375 mm (i.e., 0.016 in to 0.014 in). An optimum cutting condition produced from GA is verified with the experimental measurement. Shieh et al. [38] optimized lipase-catalyzed biodiesel production using RSM. These authors used a 5-level-5-factor central composite rotatable design to evaluate the effects on reaction time, temperature, enzyme amount, the molar ratio of methanol to soybean oil, and added water content on percentage weight conversion to soybean oil methyl ester by transesterification. In particular, the Shankar et al. [39] study indicated that RSM is an excellent method to understand process variables trends, which can either maximize or minimize product quality. The same authors also concluded that the interpretation of RSM results is very complex and often results in saddle point. Because GAs are stochastic algorithms, they can optimize complex problems where deterministic methods, such as RSM, sometimes fail.

### 1.3. Evolutionary Algorithms

**Figure 1:**
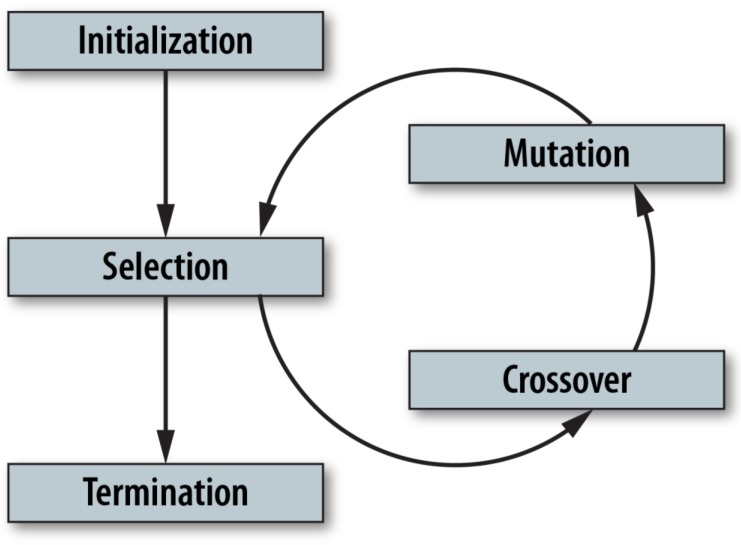
A flow diagram for a standard EA.

EAs are used to solve non-deterministic polynomial-time hardness (NP-hardness) problems and are population-based metaheuristics. They incorporate a variety of search mechanisms that use a natural selection process. EAs maintain a population of potential solutions by artificially evolving the population. The common idea behind all EAs is that given a population of individuals, environmental pressure causes them to select changes based on natural selection (i.e., survival of the fittest), which improves with every iteration. The most commonly used EA is a GA. A typical EA contains four steps: initialization, selection, genetic operators (crossover and mutation), and termination, as shown in Figure 1. GAs have gained new importance in process optimization due to their ability to solve multi-dimensional complex problems with minimum computational requirements.

In a standard GA, a population, which is a pool of possible solutions that are used as parents, is chosen randomly. The parents selected are further evaluated for their fitness. The parent with the highest fitness values are then selected for a mating routine, which is called a crossover operation. This operation results in new offspring, which are then further mutated to avoid local convergence of the algorithm. Elitism is another operator often used in a GA to make sure that the best candidates are preserved and are used in the next generation. Once the fitness does not change after a certain number of these iterations, the GA is stopped. Using this method, researchers have been able to develop new programs to solve complex problems as compared to conventional programs, which use brute force to solve optimization problems [40]. According to April et al. [41], an EA is capable of exploring the search space more thoroughly in a shorter amount of time as compared to locally based search methods, such as simulated annealing or other gradient-based approaches. Another major advantage of EAs is that unlike normal GAs, they do not require good starting points.

However, one of the major limitations of using GAs is that due to its heuristic nature, this method seldom reaches a global optimum. Therefore, GAs have difficulty solving complex variant problems [42]. These authors conclude that when GAs are used for variant problems, they result in poor fitness and bad chromosome generation. They also suggest that GA hybridization with the gradient-based method results in better conversion. They developed a hybrid genetic algorithm (HGA) and a user-friendly software tool with MATLAB. They tested the algorithm performance on the Ackley benchmark function and other food and bioengineering processes and found that the new hybrid algorithm to conventional optimization methods [42].

### 1.4. Objective

The overall objective of this present research was to understand how the grinding process variable impacts feedstock quality attributes and energy consumption of the process. Other objectives included an understanding of how grinding process variables (i.e., feedstock moisture content and grinder speed) impact quality attributes—such as bulk and tapped density, geometric mean particle length, and specific energy consumption—when a 25.4 mm (i.e., 1 in) corn stover grind was further size-reduced in a Wiley mill fitted with a 2 mm (i.e., 0.08 in) screen. The research is focused on modeling the grinding and further optimizing process variables, which could result in a maximum of the bulk and tapped density and minimize geometric mean particle size, grind moisture, and specific energy consumption.

## 2. Materials and Methods

### 2.1. Feedstock

**Figure 2:**
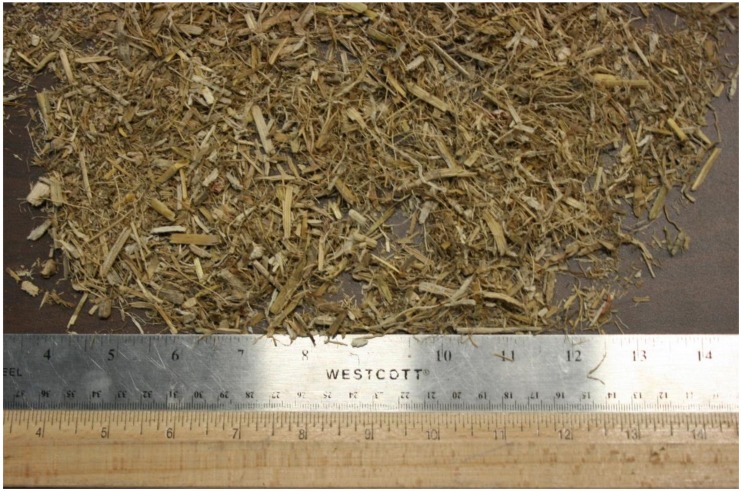
The corn stover after Stage-1 grinding.

**Figure 3:**
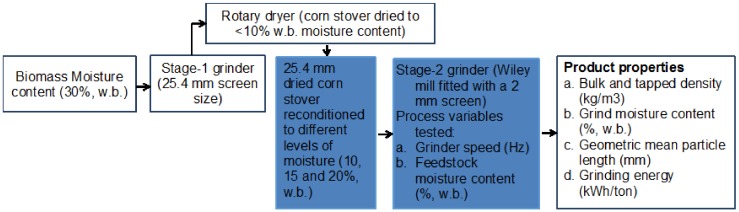
A flow diagram of the grinding studies conducted in the present research.

Corn stover that was harvested in 2014 from Story County in the State of Iowa was the feedstock used in this study. A multi-pass system was used to harvest the corn stover. The harvested biomass was further processed through a Stage-1 grinder (Vermeer HG-200 model), fitted with a 25.4 mm (i.e., 1 in) screen, and further dried in a rotary dryer to <10% (w.b.), as shown in Figure 2. This ground material was further reconditioned to different levels of moisture to understand the effect of moisture content on the Stage-2 grinding process. Figure 3 provides a flow diagram of the corn stover preprocessing, while the highlighted boxes in the figure indicate the Stage-2 grinding details that followed in this research.

### 2.2. Grinder

**Figure 4:**
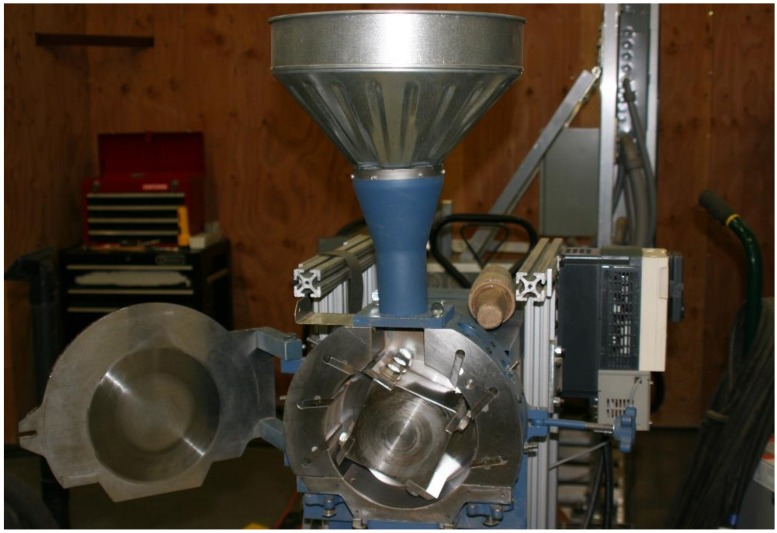
The laboratory scale Wiley mill with blades exposed.

**Figure 5:**
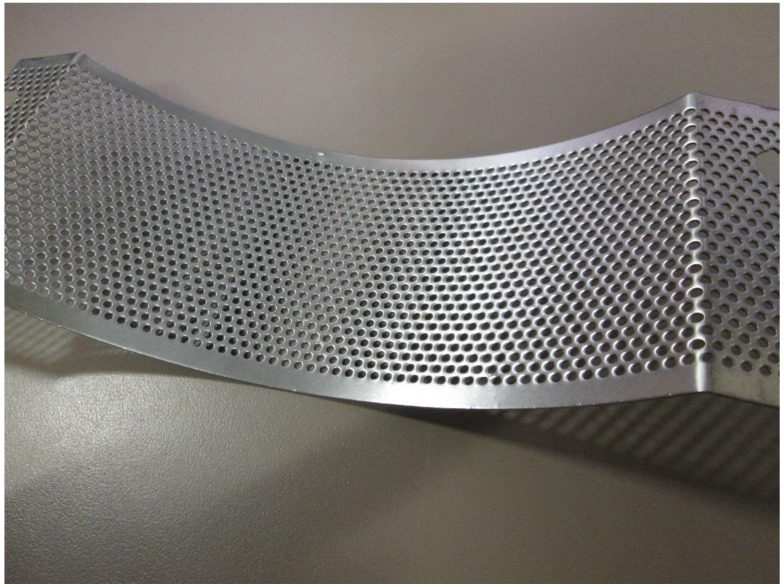
The two-mm screen used in the present study.

**Figure 6:**
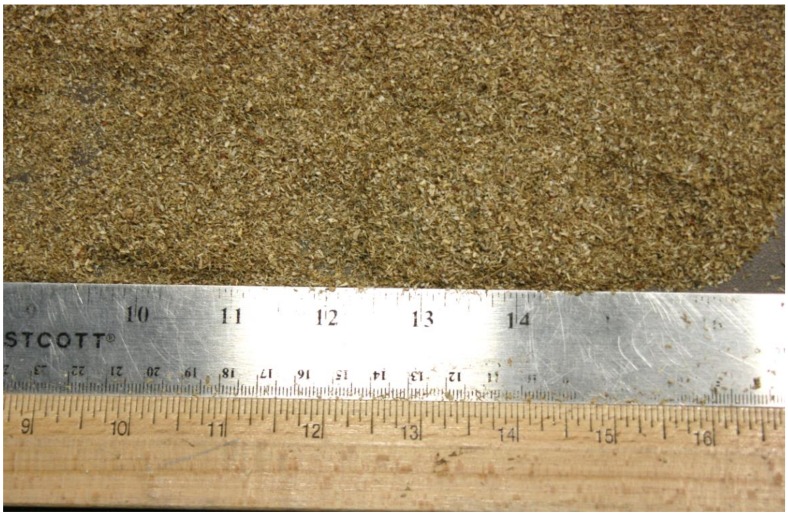
The corn stover after passing through the Wiley mill.

The grinder used for the experiment was the Wiley Laboratory Mill, a lab-scale grinder, which is shown in Figure 4. The mill contains a circular chamber lined with six stationary knives. The inside grinder chamber diameter is 19.7 cm (i.e., 7.75 in). A motor spins the inner rotor, equipped with four additional knives. The LabVIEW interface of the machine allows for the operator to adjust the speed. The rotational speed of the rotor at 60 Hz is 800 rpm. For the experiment, each sample was slowly and uniformly fed manually into the top of the machine through a funnel and then run until as much of the sample as possible had fallen through the 2 mm (i.e., 0.08 in) mesh screen (see Figure 5) at the bottom of the chamber into a container below. The surface area of the screen is 97.4 cm^2^ (i.e., 38.35 square inch) and the hole area on the grinder screen is 35.5 cm^2^ (i.e., 13.98 square inch). Once the material passes through the screen, it is then further tested for physical properties (i.e., bulk and tapped density and geometric mean particle size). The power data collected for each run was further used for grinding energy. Figure 6 shows the grind corn stover after passing through the Wiley mill.

### 2.3. Experimental Design

#### 2.3.1. Independent Variables

Two main factors—grinder speed and moisture content—were chosen for this research, as shown in Table 1. The design chosen for the experiment was a central composite design, as shown in Table 2. A two-dimensional face-centered design was chosen to understand the significance of grinder speed and moisture content on the physical properties and energy consumption. The trials that were run were initially grouped by moisture content and then performed in a random order within those groups. These measurements at strategic factor levels could be used later to model the behavior of the material under a more descriptive range of conditions.

##### Raw Material Preparation

To set the moisture content of the sample, the original pre-ground corn stover was measured to find its initial moisture content by taking small amounts of the material and then placing that material in small aluminum trays. The trays were weighed with and without the sample and then placed in an oven heated to 105 °C overnight. The trays were then weighed again, and the moisture content was calculated using Equation (1). The sample was then hydrated by adding the appropriate amount of water, as determined by Equation (2), and thoroughly mixing the sample. Finally, the moisture content was again measured for the sample to assess if the moisture content matched the desired level. About 2 kg of the 25.4 mm (i.e., 1 in) screen size with calculated amounts of water was mixed in a ribbon blender (Model: RB 500, Colorado Mill Equipment, Canon City, CO, USA) to adjust the moisture content to the desired levels based on the experimental design, as shown in Table 2. Each batch of the mixed sample was divided into three parts to conduct the grinding experiments. Once the grinding was done, the three samples were further used to measure the grind physical properties.

(1) $$Moisture\;Content\;\left(\%\right)=\;\frac{Mass\;of\;moist\;sample\;\left(g\right)-\;mass\;of\;dry\;sample\;\left(g\right)}{Mass\;of\;wet\;sample\;\left(g\right)}\times 100$$

(2) $${\mathrm{Weight}}_{\mathrm{add}}=\frac{{\mathrm{Weight}}_{\mathrm{initial}}({\mathrm{MC}}_{\mathrm{final}}-{\mathrm{MC}}_{\mathrm{initial}})}{1-{\mathrm{MC}}_{\mathrm{final}}}\times (1+\mathrm{adjustment}\;\mathrm{factor})$$

##### Grinder Speed (Hz)

The Wiley mill used in the experiment had the capability to adjust the machine speed via a LabVIEW user interface. This device was used to adjust the speed to 20, 40, or 60 Hz for each experiment, as necessary.

#### 2.3.2. Dependent Variables

Five dependent variables were measured in the experimental process—specific energy consumption, grind moisture content, particle size distribution, bulk density, and tapped density. For each of these dependent variables, all measurements were made immediately after grinding to understand the effect of the grind moisture impact on the bulk density. All the measurements were repeated three times to account for the variability that is likely to occur with the experimental results.

##### Bulk and Tapped Density

The bulk and tapped density were calculated per American Society of Agricultural and Biological Engineers (ASABE) standards [43] using a cylindrical sample container. The container was filled with the sample, leveled, and then weighed by subtracting the weight of the empty container. The container was then tapped five times by dropping it from a height of around 152.4 mm (i.e., 6 in) onto a flat surface. Afterward, the container was topped off, leveled, and then weighed again. The sample weights before and after tapping are divided by the volume of the interior of the container to determine the bulk and tapped densities, as given in Equation (3).

(3) $$Bulk\;\left(Tapped\right)\;Density\left(\frac{kg}{m^{3}}\right)=\frac{Mass\;of\;sample\;\left(kg\right)}{volume\;of\;cylinder\;\left(m^{3}\right)}$$

##### Moisture Content

The final moisture content was calculated by recording the weight of several small samples before and after drying them overnight in an oven, again calculated using Equation (1).

##### Particle Size Distribution

For particle size distribution, the rest of the sample was put into an oven heated at 105 °C for about an hour so that the material could be dry for using the Ro-Tap. The Ro-Tap contained seven meshes between sizes 12 and 230, in addition to the pan at the bottom, and was run for 1 h. The mean particle size was then calculated using American National Standards Institute/American Society of Agricultural Engineers (ANSI/ASAE) standards [44]. The particle size distribution data was further used to calculate the geometric mean particle length of the corn stover grind.

##### Specific Energy Consumption (SEC)

The machine power was recorded automatically during the experiment using LabVIEW. The average power, grinding time, and final sample weight was then used to find the specific energy consumption in kWh/ton, as given in Equation (4).

(4) $$SEC\;\left(\frac{kW\times hr}{kg}\right)=\frac{Full\;load\;power\;\left(kW\right)-No\;load\;power\;\left(kW\right)\times time\;\left(hr\right)}{mass\;of\;sample\;\left(ton\right)}.\;$$

##### Statistical Analysis of the Experimental Data

The standard deviation calculations for each of the response variables were performed in Microsoft Excel. The experimental data that is collected using the central composite design was further used to develop the response surface models and surface plots, which were developed using Statistica 9.1 [45]. The response surface plots were drawn to understand the interactive effect of the process variables (i.e., grinder speed and feedstock moisture content) on the grind physical properties (e.g., bulk and tapped density, geometric mean particle size, and moisture content) and specific energy consumption of the grinding process.

The hybrid genetic algorithm (HGA) used in the present study has two optimization routines: GA and gradient-based method. As GAs, are heuristic nature, they do not help to reach the global optimum. So Tumuluru and McCulloch [42] hybridized a GA with a gradient-based method for better conversion. These authors developed this algorithm and a user-friendly software tool on MATLAB, further tested it on the optimization problems, and concluded that a HGA helps to converge at the optimum values more precisely compared to the regular GA. More details about the algorithm used in the present study can be found in the research completed by Tumuluru and McCulloch [42] on the optimization of food and bioengineering processes.

In the case of optimization of the grinding process, the response surface models that are developed using the experimental data in Table 2 were further used as the objective functions. These objective functions are either minimized or maximized using the hybrid GA. In the case of bulk and tapped density, the objective functions were maximized, whereas in the case of geometric mean particle length, grind moisture content, and specific energy consumption, the objective functions were minimized to find the optimum process conditions. To find the common optimum process conditions, which can result in a maximum of bulk and tapped density and a minimum of geometric mean particle length, grind moisture content, and specific energy consumption, the method developed by Shankar and Bandyopadhyay [46] was used. According to this method, a combined model was developed using the regression equations developed for the extrusion process. For a maximization problem, the equation needing to be maximized was used as such, whereas a minus sign was added to the equations needing to be minimized. When the combined function has optimized, the equations with a positive sign result in maximum values, whereas the equations with a negative sign result in minimum values. In the present study, a positive sign is used for bulk and tapped density equations, whereas geometric mean particle length, grind moisture content, and specific energy consumption equations were given a negative sign. Equations (5)–(9) are used to find the individual optimum process conditions, whereas Equation (10) is used for common optimum process conditions, which can result in a maximum of bulk and tapped density and a minimum of geometric mean particle length, grind moisture content, and specific energy consumption.

##### Individual Optimum Process Condition Equations

(5) f(y)=Maximize (BD model)

(6) f(y)=Maximize (TD model)

(7) f(y)=Minimize (GMPL model)

(8) f(y)=Minimize (GMC model)

(9) f(y)=Minimize (SEC model)

##### Common Optimum Process Condition Equation

(10) f(y)=Maximize ((BD+TD model)−(GMPL model+GMC model+SEC model))

Note: BD: Bulk density (kg/m^3^); TD: Tapped density (kg/m^3^); GMPL: Geometric mean particle length (mm); GMC: Grind moisture content (%, w.b.); SEC: Specific energy consumption (kWh/ton).

## 3. Experimental Results

**Figure 7:**
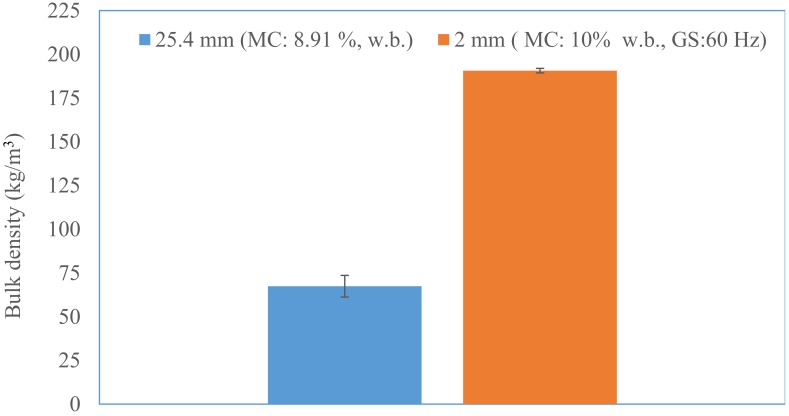
The comparison of the bulk density of the 25.4 mm and 2 mm screen size grind. (Note: GM: grinder speed and MC: corn stover moisture content).

**Figure 8:**
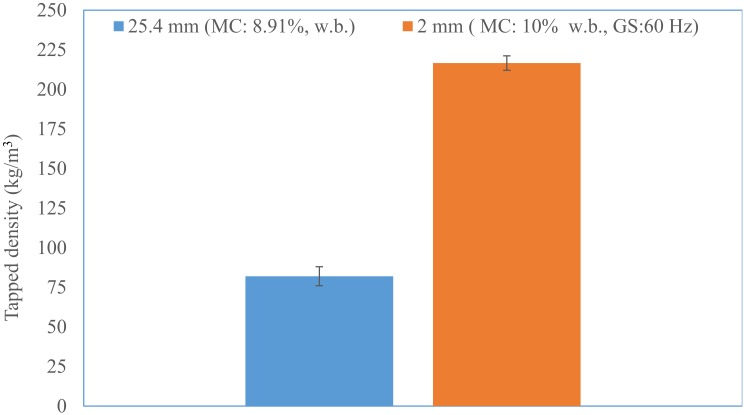
The comparison of the tapped density of the 25.4 mm and 2 mm screen size grind. (Note: GM: grinder speed and MC: corn stover moisture content).

**Figure 9:**
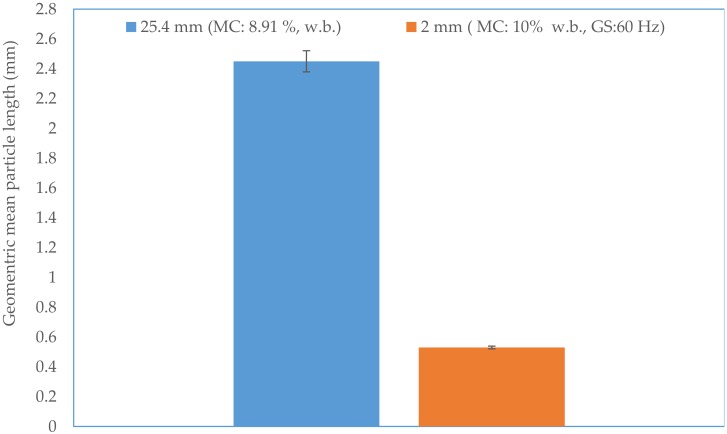
The comparison of geometric mean particle length of the 25.4 mm and 2 mm screen size grind. (note: GM: grinder speed and MC: corn stover moisture content).

The initial moisture content of the 25.4 mm (i.e., 1 in) hammer milled corn stover was about 8.91% (w.b). The reconditioned moisture values of the 1 in corn stover at 10, 15, and 20% (w.b.) moisture content were 10.12% (sd: 0.29), 14.81% (sd: 0.06), and 19.93% (sd: 0.29). The bulk density and tapped density of 25.4 mm (i.e., 1 in) grind corn stover was about 67.39% (sd: 6.2) and 82.01% (sd: 6.0) kg/m^3^. It is clear from Figure 7 and Figure 8 that the bulk and tapped density increases by almost 2.6 to 2.8 times when corn stover is ground with a hammer mill fitted with a 25.4 mm screen size, which is further size-reduced using a Wiley mill fitted with a 2 mm (i.e., 0.08 in) screen size at about a 10% moisture content. The bulk and tapped density observed after grinding in the Wiley mill was about 190 and 216 kg/m^3^. Similarly, the mean particle size-reduced significantly by about 4.6 times when ground in a Wiley mill fitted with a 2 mm (i.e., 0.08 in) screen size, which represents a reduction from 2.45 mm to 0.53 mm (0.09 in to 0.02 in), as shown in Figure 9. Table 3 indicates the experimental results obtained based on the central composite experimental design given in Table 2.

### 3.1. Response Surface Models and Plots

The experimental data was further used to fit the response surface models, as shown in Table 4. The coefficient of determination values of the models developed indicated that they have adequately described the grinding process tested. These RSM models were further used to develop surface plots to understand the interaction effect of the process variables on the product properties and specific energy consumption and further optimize the process to identify the process conditions, which can maximize the bulk and tapped density and minimize geometric mean particle length, grind moisture content, and specific energy consumption.

#### Response Surface Plots

**Figure 10:**
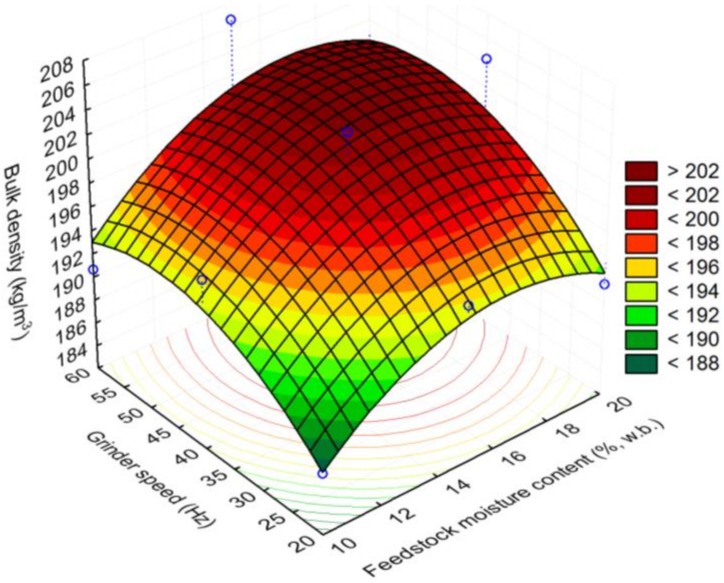
The effect of feedstock moisture content and grinder speed on bulk density.

**Figure 11:**
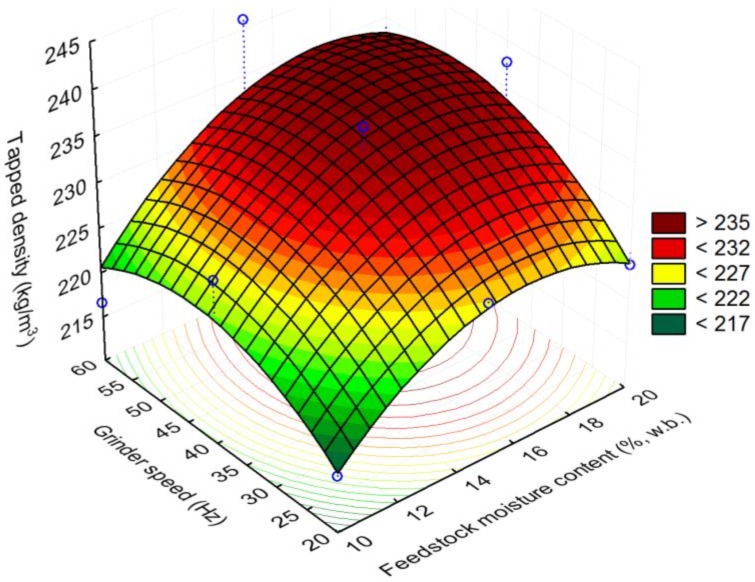
The effect of feedstock moisture content and grinder speed on tapped density.

Response surface plots were drawn using the response surface models that are developed for the process variables grinder speed (Hz) and feedstock moisture content (% w.b.). The bulk density plot indicated that with an increase in feedstock moisture content to 20% (w.b.) and the grinder speed to 60 Hz, the bulk density values increased to >202 kg/m^3^, whereas lowering the feedstock moisture content to 10% (w.b.) and the grinder speed to about 20 Hz reduced the bulk density to <188 kg/m^3^ (see Figure 10). The surface plot also indicated that at a lower grinder speed of 20 and 25 Hz and increasing the feedstock moisture content increased the bulk density values from 184–192 kg/m^3^, whereas increasing the grinder speed to 60 Hz did increase the bulk density values further. The trends of the surface plot drawn for tapped density were similar to bulk density observations, where a higher grinder speed of 60 Hz and a higher feedstock moisture content of 20% (w.b.) increased the tapped density values to >235 kg/m^3^ (see Figure 11). At a lower grinder speed of 20 Hz and a lower feedstock moisture content of 10% (w.b.), the tapped density values observed were <217 kg/m^3^. The tapped density increased to >232 kg/m^3^ at a feedstock moisture content of >14% (w.b.) and a grinder speed of >40 Hz.

**Figure 12:**
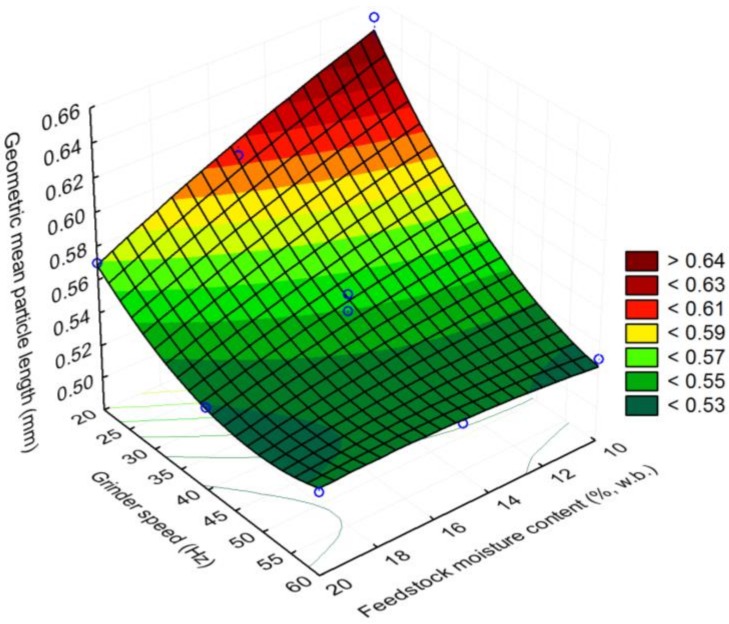
The effect of feedstock moisture content and grinder speed on geometric mean particle length.

In the case of geometric mean particle length, a higher grinder speed and a lower moisture content lowered the geometric mean particle size. At a 60 Hz grinder speed and 10% (w.b.) moisture content, the geometric mean particle size observed was <0.53 mm, whereas at 20% (w.b.) feedstock moisture content at 60 Hz, the geometric mean particle length was in the range of 0.53 to 0.55 mm, as shown in Figure 12. Increasing the feedstock moisture content had a marginal effect on the geometric mean particle size. At a lower grinder speed of 20 Hz and a feedstock moisture content in the range of 10–12% (w.b.), the geometric mean particle size observed was in the range of 0.61 to 0.64 mm. It can be concluded from the surface plot that at a lower grinder speed, the feedstock moisture content had a great effect, whereas, at a higher grinder speed, the feedstock moisture content effect was marginal.

**Figure 13:**
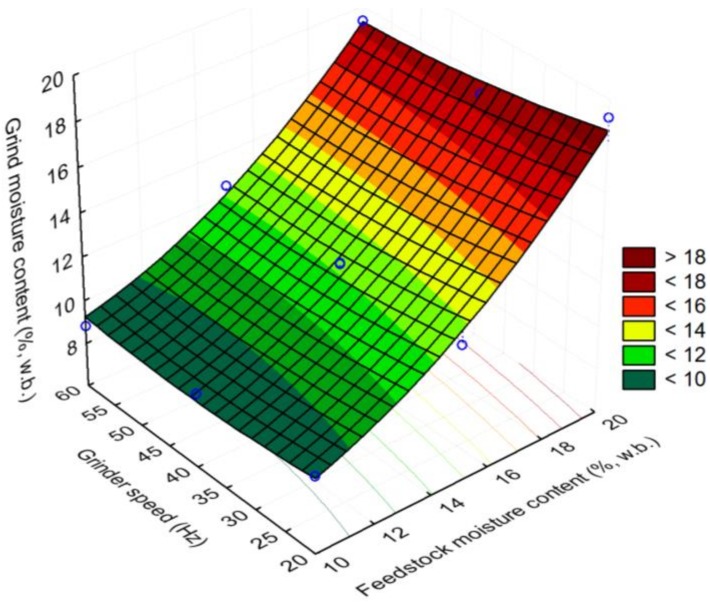
The effect of feedstock moisture content and grinder speed on grind moisture content.

In the case of grind moisture content, the initial moisture content played a major role on the final moisture content of the grind. The results indicated that there is about a 2% (w.b.) moisture loss during grinding. The moisture loss during grinding was higher at a higher feedstock moisture content as compared to a lower feedstock moisture content, as shown in Figure 13. At a lower feedstock moisture content of 10%, the final moisture content of the observed grind was about 9% (w.b.), whereas, at a higher feedstock moisture content of 20% (w.b.), the final moisture content of the grind was about 17.5% (w.b.). Additionally, the surface plot indicated that grinder speed had a marginal effect on the moisture loss in the corn stover grind. At a 10% (w.b.) moisture content and a 20 Hz grinder speed, the moisture loss was about 0.5% (w.b.), whereas, at 60 Hz grinder speed, the moisture loss in the corn stover grind was about 1.2% (w.b.). Additionally, at a higher moisture content of 20% (w.b.) and a 20 Hz grinder speed, the moisture loss observed in the corn stover grind was about 0.8% (w.b.), whereas increasing the grinder speed to 60 Hz resulted in about a 2.7% (w.b.) moisture loss in the corn stover grind.

**Figure 14:**
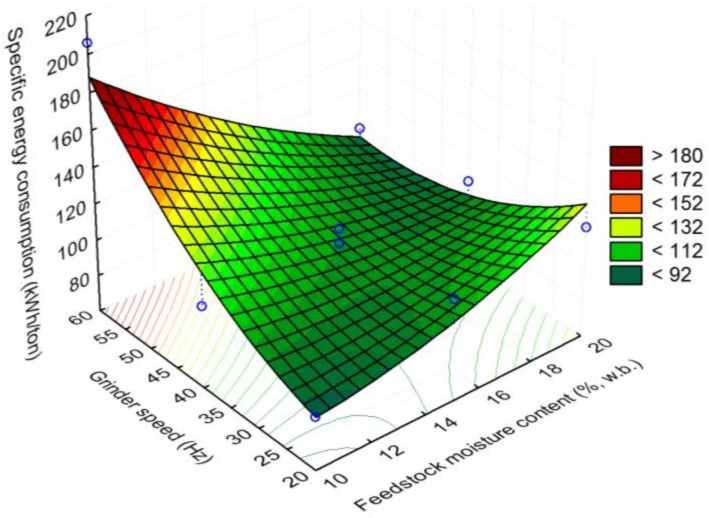
The effect of feedstock moisture content and grinder speed on specific energy consumption.

In the case of specific energy consumption (kWh/ton), the lowest values of <92 kWh/ton were observed at a lower feedstock moisture content of 10% (w.b.) and a grinder speed of 20 Hz, whereas increasing the grinder speed to 60 Hz at a lower feedstock moisture content resulted in the highest specific energy consumption values of >180 kWh/ton, as shown in Figure 14. Increasing the grinder speed to 60 Hz and feedstock moisture content to 20% (w.b.) resulted in energy consumption values of about 92–112 kWh/ton.

Table 5 indicates the trends of the process variables that can result in the maximization of bulk and tapped density (kg/m^3^) and a minimum of geometric mean particle length (mm), grind moisture content (% w.b.), and specific energy consumption (kWh/ton). It is clear from the table that medium-to-higher grinder speeds (Hz) and feedstock moisture content (% w.b.) resulted in the maximization of bulk and tapped density. In the case of grind moisture content (% w.b.), the lower feedstock moisture content of 10% and lower-to-higher grinder speed resulted in a lower grind moisture content. In the case of geometric mean particle length, lower and higher feedstock moisture contents (% w.b.) and medium-to-higher grinder speeds resulted in a lower geometric mean particle length. The specific energy consumption of the grinding process was found to be lower at lower-to-medium feedstock moisture contents (% w.b.) and lower grinder speeds (Hz).

### 3.2. Optimization

The response surface plots could not indicate the process conditions (i.e., grinder speed and feedstock moisture content), which can maximize the bulk and tapped density and minimize geometric mean particle length and grinder energy consumption. The hybrid GA developed by Tumuluru and McCulloch [42] was used for optimization to identify the process conditions that can maximize the bulk and tapped density and minimize the geometric mean particle size and grinding energy consumption. The details of the hybrid genetic algorithm (HGA) that was used in the present study are explained in Section 2.3.2. Table 6 indicates the individual and common optimized process conditions identified using a HGA. It is very clear from the table that a maximum bulk density of about 203 kg/m^3^ can be obtained at a grinder speed of 49.65 Hz and feedstock moisture content of 17.04% (w.b.), whereas in the case of tapped density, a maximum value of about 237 kg/m^3^ can be obtained at a grinder speed of 47.61 Hz and a feedstock moisture content of 17.14% (w.b.). The geometric mean particle length of 0.526 mm was predicted at a grinder speed of 48.60 Hz and a feedstock moisture content of 19.76% (w.b.), whereas a minimum grind moisture content of 9.04% was predicted at a grinder speed of 43.33 Hz and 10.65% (w.b.) feedstock moisture content. In the case of specific energy consumption, a minimum value of 89.72 kWh/ton was observed at a grinder speed of 20.18 Hz and feedstock moisture content of 10.34% (w.b.). In the case of common optimum process conditions, a feedstock moisture content of 19.51% (w.b.) and grinder speed of 50.63 Hz predicted the maximum bulk and tapped density values of about 201.6 and 235.36 kg/m^3^ and the minimum geometric mean particle length, grind moisture content, and specific energy consumption values of about 93.36 kWh/ton, 0.527 mm, and 16.78% (w.b.).

## 4. Discussion

In the present study, the physical properties and bulk and tapped densities were maximized, while the geometric mean particle length was minimized at a medium-to-high moisture content and grinder speed. In general, the bulk density of the ground material is a function of moisture content, screen size, particle size, particle size distribution, and particle aspect ratio. Tumuluru and Yancey [7] indicated that both grinder type and the type of feedstock and feedstock moisture content impacted the bulk density of the biomass. Their study also indicated that vortex mills resulted in a higher bulk density as compared to rotary shear and hammer mills. In the case of energy consumption, a vortex mill consumed the maximum amount of grinding energy, which is followed by a hammer mill and rotary shear mill. In the case of mean particle size, the rotary shear resulted in maximum particle size, followed by hammer and vortex mills. In this study, both grinder speed and corn stover grind moisture content influenced the bulk and tapped density. The response surface plots and the optimized conditions identified in the present study indicated that medium to high corn stover grind moisture content and grinder speed maximized the bulk and tapped density. Kaliyan et al. [47] found that an increase in screen size results in an increase in geometric mean length of particles and throughput, but also a decrease in bulk density of the particles and specific energy consumption. According to Pfost and Headley [48], the speed has a significant effect on the mean particle size, which corroborates with the present study where both feedstock moisture content and grinder speed have an impact on the geometric mean particle length. The optimized process conditions indicated that a higher feedstock content of 19.51% and 50.63 Hz resulted in the minimum geometric mean particle length. Balk [49] pointed out that at slower speeds, the material impinges on the screen at a greater angle causing greater amounts of coarser feed to pass through. This observation has matched with the present study where a lower grinder speed resulted in larger geometric mean particle length.

The present research indicated that both feedstock moisture content and grinder speed had a great effect on the specific energy consumption. It is clear from the present research that lowering the feedstock moisture content and lowering the grinder speed reduced the specific energy consumption of the size-reduction process. The lower energy consumption values are observed at a lower moisture content of 10% (w.b.) and a lower grinder speed of 20 Hz. Bitra et al. [27] found that the total specific energy for agricultural biomass chopping increases with knife mill speed. This result corroborates with the present energy consumption data, where increasing the grinder speed increased the specific energy of the grinding process. The study conducted by Tumuluru and Yancey [7] indicated that moisture in biomass has a significant effect on grinding energy and product quality. Their study also indicated that increasing moisture content increased the particle size, but other factors, such as grinder speed, feed rate, and screen size, also affect the grinding energy and physical properties of the ground material. These results have corroborated with the present finding where both feedstock moisture content and grinder speed had an impact on the grind properties and specific energy consumption of the process. Mani et al. [22], Balk [48], and Souček et al. [50] found a positive correlation between the moisture content and the specific energy consumption of agricultural biomass, which has matched with the trends observed in the present research where at lower feedstock moisture content, the specific energy consumption was lower. According to Mani et al. [22], moisture content had a positive correlation with the specific energy consumption of wheat and barley straws, corn stover, and switchgrass, the higher the moisture content, the higher the specific energy consumption. This observation has matched with the present study where the optimized process conditions indicated that a lower moisture content of 10% (w.b.) and a lower grind speed of about 20 Hz resulted in lowest specific energy consumption values. These results have corroborated with Fitzgerald and Themelis [51] study where the size-reduction of municipal solid waste (MSW), was optimized by lowering the rotor speed. Studies conducted at Idaho National Laboratory on understanding the effect of grinder speed, and moisture content on grinding energy consumption of a commercial scale Stage-1 grinder (Vermeer, Pella, Iowa, Model No: BG480E) indicated that at a lower moisture content of 13.2% (w.b.) and with a 60 Hz grinder speed, the energy consumption was about 12.58-kWh/dry ton, whereas by increasing the moisture content to 26.3% (w.b.) for the same grinder, the speed decreased the grinding energy consumption to 11.86-kWh/dry ton [52]. This observation has matched with our current study where increasing the grinder speed at a higher moisture content helped to reduce the specific energy consumption of the grinding process. This study could not clearly indicate why the grinding energy decreased with increase in grinder speed at a higher moisture content, but we think further studies on the impact of screen size and feed rate on energy consumption will help to understand how different processes can impact the grinding energy and grind properties.

## 5. Conclusions

This present study indicated that both grinder speed and corn stover moisture content had an impact on the grind physical properties and specific energy consumption of the process. Based on this present study, the following conclusions have been drawn:

1. The initial bulk density and tapped density of 25.4 mm (i.e., 1 in) grind corn stover was about 67.39% (sd: 6.2) and 82.01% (sd: 6.0) kg/m^3^, when ground in a Wiley mill fitted with a 2 mm (i.e., 0.08 in) screen at different grinder speeds and moisture contents; the bulk and tapped density were in the range of 188–202 and 217–235 kg/m^3^.
2. Response surface models developed for the experimental data using the central composite design for the corn stover grinding adequately described the process based on the coefficient of the determination values.
3. The response surface plots indicated that a higher moisture content and higher grinder speed increased the bulk and tapped density and minimized the geometric mean particle length. The grind moisture content was minimized when the initial moisture content of corn stover was lower and specific energy consumption decreased at lower moisture content and lower grinder speed.
4. Optimization of the process using hybrid GA indicated that a higher moisture content of 17–20% (w.b.) and a higher grinder speed of 47–50 Hz maximized the grind physical properties such as bulk and tapped density (201 and 235 kg/m^3^), and minimized the geometric mean particle length (0.53 mm). In the case of the grind moisture content, the initial moisture content of the corn stover played a major role on the final grind moisture content, whereas the grinder speed had a marginal effect.
5. In the case of specific energy consumption, a minimum value of 93 kWh/ton was predicted at a lower moisture content of 10% (w.b.) and a lower grind speed of 20 Hz.

## Figures and Tables

**Table 1:** The levels for the two factors.

	Grinder Speed (Hz) (x_1_)	Feedstock Moisture Content (%, w.b.) (x_2_)
Low	20	10
Medium	40	15
High	60	20

**Table 2:** The central composite design with coded and descriptive levels.

Expt. No.	x_1_	x_1_	Corner	Grinder Speed (Hz)	Feedstock Moisture Content (%, w.b.)
1	0	0	ab/2	40	15
2	−1	−1	“(1)”	20	10
3	1	−1	a	60	10
4	−1	1	b	20	20
5	1	1	ab	60	20
6	0	0	ab/2	40	15
7	0	−1	a/2	40	10
8	−1	0	b/2	20	15
9	0	1	a/2, b	40	20
10	1	0	b/2, a	60	15

**Table 3:** The experimental data based on the central composite design.

Expt. No.	Bulk Density (kg/m^3^)	SD	Tapped Density (kg/m^3^)	SD	Geometric Mean Particle Length (Xgm) (mm)	SD	Grind Moisture Content (%, w.b.)	SD	Specific Energy Consumption (kWh/ton)	SD
7	196.3	3.9	228.0	3.3	0.55	0.009	9.37	0.17	105.1	7.2
2	187.2	7.5	216.2	6.1	0.65	0.018	9.51	0.27	89.4	18.5
3	190.6	1.2	216.5	4.5	0.53	0.009	8.80	0.44	205.8	25.4
1	192.8	5.7	224.2	8.0	0.56	0.004	12.25	0.43	112.8	4.7
6	203.2	3.5	237.3	0.4	0.55	0.01	12.28	0.37	104.9	6.4
8	195.3	3.0	227.1	3.0	0.6	0.014	12.19	0.53	116.1	6.5
10	206.6	5.9	240.9	7.6	0.53	0.005	12.44	0.45	110.1	5.0
9	204.3	4.3	237.7	1.5	0.53	0.004	17.09	0.28	106.0	8.6
5	197.0	2.7	229.4	5.1	0.53	0.01	17.36	0.53	97.7	6.2
4	191.5	1.9	223.8	1.2	0.57	0.008	19.22	0.25	120.5	17.9

**Table 4:** The response surface models.

Physical Properties and Grinding Energy	Response Surface Model	(R^2^)
Bulk Density (kg/m^3^)	134.04 + 5.66x_1_ + 0.840x_2_ − 0.1747$x_{1}^{2}$ − 0.00935$x_{2}^{2}$ + 0.005014x_1_x_2_	0.60
Tapped Density (kg/m^3^)	144.43 + 7.9975x_1_ + 0.9876x_2_ − 0.2507$x_{1}^{2}$ − 0.01276$x_{2}^{2}$ + 0.013218x_1_x_2_	0.62
Geometric mean particle length (Xgm)	0.8495 − 0.007048x_1_ − 0.00920x_2_ − 0.000143$x_{1}^{2}$ + 0.000054$x_{2}^{2}$ + 0.00020x_1_x_2_	0.97
Grind moisture content (% w.b.)	10.3516 − 0.480027x_1_ − 0.035933x_2_ + 0.048745$x_{1}^{2}$ + 0.000749$x_{2}^{2}$ − 0.002891x_1_x_2_	0.99
Specific energy consumption (% w)	28.74513 + 2.604975x_1_ + 2.986682x_2_ + 0.292750$x_{1}^{2}$ + 0.037081$x_{2}^{2}$ − 0.348151x_1_x_2_	0.82

Note: x_1_: Feedstock moisture content (%, w.b.); x_2_: Grinder speed (Hz); R^2^: Coefficient of determination.

**Table 5:** The trends of the process variables based on RSM.

	Predicted	Predicted	x_1_ (Feedstock Moisture Content (% w.b.)	x_2_ (Grinder Speed) (Hz)
Bulk Density (kg/m^3^)	Max	>202	14–20	40–60
Tapped Density (kg/m^3^)	Max	>232	14–20	40–60
Grinding Energy Consumption (kWh/ton)	Min	<92	10–15	20–40
Geometric Mean Particle Length (mm)	Min	<0.53	10–20	40–60
Grind Moisture Content (% w.b.)	Min	<9	10	20–60

**Table 6:** The optimized process conditions obtained using a hybrid genetic algorithm (HGA).

			Individual Optimum Process Conditions	
	Predicted (Maximum)	Predicted (Minimum)	x_1_ (Feedstock Moisture Content (% w.b.)	x_2_ (Grinder Speed) (Hz)
Bulk Density (kg/m^3^)	202.81		17.04	49.65
Tapped Density (kg/m^3^)	236.72		17.14	47.61
Grinding Energy Consumption (kWh/ton)		89.72	10.34	20.18
Geometric Mean Particle Length (mm)		0.526	19.76	48.60
Grind Moisture Content (% w.b.)		9.04	10.65	43.33
			**Common Optimum Process Conditions**	
	**Predicted (Maximum)**	**Predicted (Minimum)**	**x_1_ (Feedstock Moisture Content (% w.b.)**	**x_2_ (Grinder Speed)(Hz)**
Bulk Density (kg/m^3^)	201.61		19.51	50.63
Tapped Density (kg/m^3^)	235.36			
Grinding Energy Consumption (kWh/ton)		93.36		
Geometric Mean Particle Length (mm)		0.527		
Grind Moisture Content (% w.b.)		16.78		

## U.S. Department of Energy Disclaimer

This information was prepared as an account of work sponsored by an agency of the U.S. Government. Neither the U.S. Government nor any agency thereof, nor any of their employees, makes any warranty, express or implied, or assumes any legal liability or responsibility for the accuracy, completeness, or usefulness of any information, apparatus, product, or process disclosed, or represents that its use would not infringe privately owned rights. References herein to any specific commercial product, process, or service by trade name, trademark, manufacturer, or otherwise, does not necessarily constitute or imply its endorsement, recommendation, or favoring by the U.S. Government or any agency thereof. The views and opinions of authors and editor expressed herein do not necessarily state or reflect those of the U.S. Government or any agency thereof.

## Nomenclature

ANSI	American National Standards Institute
ASABE	American Society of Agricultural and Biological Engineers
ASAE	American Society of Agricultural Engineers
BETO	Bioenergy Technologies Office
DOE	U.S. Department of Energy
EA	Evolutionary Algorithms
FAO	Food and Agricultural Organization
GA	Genetic Algorithm
HGA	Hybrid Genetic Algorithm
Hz	Hertz
in	inches
RSM	Response Surface Methodology
w.b.	Wet Basis
μm	Micrometer
